# Does pulmonary rehabilitation alter patients’ experiences of living with chronic respiratory disease? A qualitative study

**DOI:** 10.2147/COPD.S165623

**Published:** 2018-08-08

**Authors:** Rupert Jones, Herbert Muyinda, Grace Nyakoojo, Bruce Kirenga, Winceslaus Katagira, Jillian Pooler

**Affiliations:** 1 Clinical Trials and Population Studies, Plymouth University Peninsula Schools of Medicine and Dentistry, Plymouth, UK, rupert.jones@plymouth.ac.uk; 2 Makerere University Lung Institute, College of Health Science, Makerere University, Kampala, Uganda

**Keywords:** exercise therapy, non-pharmacological treatment, self-management, stigma

## Abstract

**Purpose:**

Chronic respiratory disease (CRD) including COPD carries high and rising morbidity and mortality in Africa, but there are few available treatments. Pulmonary rehabilitation (PR) is a non-pharmacological treatment with proven benefits in improving symptoms and exercise capacity, which has not been tested in Africa. We aimed to evaluate the lived experience of people with CRD, including physical and psychosocial impacts, and how these are addressed by PR.

**Patients and methods:**

A team of respiratory specialists, nurses, and physiotherapists implemented PR to meet the clinical and cultural setting. PR consisted of a 6-week, twice-weekly program of exercise and self-management education. Forty-two patients were recruited. Qualitative data were collected through interviews with patients at baseline and six weeks post-completion, focus group discussions, ethnographic observations, and brief interviews.

**Results:**

Before and after PR, a total of 44 semi-structured interviews, 3 focus group discussions, and 4 ethnographic observations with brief interviews were conducted. Participants reported profound problems with respiratory symptoms, functional impairment, wide-reaching economic and psychological impacts, and social isolation. Patients who were debilitated by their condition before PR reported that PR addressed all their major concerns. It was reported that breathlessness, pain, immobility, weight loss, and other CRD-related symptoms were reduced, and social and intimate relationships were improved. Local materials were used to improvise the exercises, enabling some to be maintained at home. Recommendations for future PR programs included patient information to take home as a reminder of the exercises, and to show their families, and the support of a community health worker to help maintenance of exercises at home.

**Conclusion:**

PR has the potential to restore the physical, mental, and social functioning in patients with CRD, whereas medication has much more narrow effects. PR offers a major new option for treatment of a neglected group of patients.

## Introduction

Chronic respiratory diseases (CRDs), including COPD and post-tuberculosis lung damage (p-TBLD), are an increasing problem globally^1^ and COPD is now the third leading cause of death worldwide.^2^ In Africa, CRDs are rising due to colliding epidemics of tobacco smoking, HIV, and exposure to air pollution from biomass smoke and outdoor air pollution.^3^ Estimates of the prevalence of COPD vary, but in well-conducted surveys, the prevalence in South Africa was 23%^4^ and Uganda 16%.^5^ A systematic review estimates that the prevalence has risen by 35% in a decade^6^ and is more prevalent in the poorest populations.^7^ P-TBLD is also a growing and poorly understood condition.^8^ In many patients with tuberculosis (TB), despite bacterial cure, there are ongoing symptoms related to the severe irreversible lung damage inflicted by the infection.^9^

Whether from COPD or p-TBLD, people with CRD typically experience problems of daily symptoms of breathlessness, cough, and fatigue, punctuated by acute exacerbations.^1^ The symptoms often affect ability to work or function normally, impacting on the patient, their families, and health services.^1^,^2^,^10^,^11^ The health deficits reach well beyond the lungs; psychosocial impacts and stigma may be profound.^12^ While in Western countries a range of drug treatments may alleviate symptoms and reduce risks of exacerbations, these are long-term treatments which are seldom available or affordable in sub-Saharan Africa. For many in Africa, there are no effective treatments and new treatment options, such as pulmonary rehabilitation (PR), are needed to address the rising burden of CRDs on individuals, society, and the health services.^13^

PR is an intervention involving physical exercises to address deconditioning and reduce breathlessness and fatigue and provide education about CRDs and how patients can manage the symptoms themselves.^14^ The intervention addresses physical incapacity, demotivation, anxiety, depression and social isolation, and psychosocial support. PR has the potential to restore people with CRD as far as possible to their previous condition and to develop to the maximum extent their physical, mental, and social functioning.^14^,^15^ PR is as much, or more, cost-effective than pharmacological treatment for COPD^16^,^17^ and addresses a wide range of physical, psychological, behavioral, and social issues that combine to harm a patient.^17^ PR may be implemented using existing staff with minimal equipment so requires little investment and is sustainable and scalable.

While CRD is now a leading cause of death and disease, very little is known about its impact on patients’ lives in Africa. We hypothesized that, given fears created by the general lack of awareness of the CRDs among the health service and the population in Uganda, and with adverse health beliefs such as witchcraft and magic,^18^ there was scope for patient education to have profound improvement in quality of life. This qualitative study aimed to explore experiences of PR on the lives of patients with COPD in Uganda. The research sought to answer two questions: 1) what are the lived experiences of patients diagnosed with COPD? and 2) how does PR alter these experiences? A PR program was run in Mulago National Referral Hospital, Kampala, Uganda, for patients with disabling breathlessness from p-TBLD and COPD and showed good improvements in quantitative outcomes, including exercise capacity, quality of life, and biometrics.^19^

This paper reports patients’ perspectives on the way in which COPD not only caused physical symptoms but also affected psychological and social well-being and how implementing PR may address these issues.

## Patients and methods

### The PR program

Details of the design conduct and quantitative outcomes have been reported previously.^19^ In brief, participants in the PR program were recruited from the outpatient department at Mulago Hospital, Kampala and had a confirmed diagnosis of p-TBLD or COPD. Patients who fulfilled the inclusion criteria – Medical Research Council (MRC) dyspnoea score grade 2 or higher – were invited to take part in the program, and written informed consent was obtained. We excluded those with unstable cardiovascular disease and locomotor difficulties that precluded the exercise. Details of the participants’ current level of activity and health status were recorded, which helped them set goals for what was most important to each of them to achieve in the PR program.

The PR program consisted of exercises delivered by a physiotherapist, and health education delivered by the physiotherapist, doctor, nurses, dietitian, and a counselor. Patients attended twice a week for six weeks for ~2 hours per session. The physical exercises were designed to improve strength, endurance, and flexibility and included sit-to-stand, step-up, biceps curl, cycling, pull-ups, and walking.

The intervention was performed in groups, but the exercise training was individually prescribed and intensified as the program progressed. Participants learnt about breathing-related complications and techniques of ameliorating their effects, the necessary (and unhelpful) medications, nutrition and balanced diet, relaxation and the need to avoid stress, how to go about their everyday tasks without much difficulty, and how to cope with and manage the changes that often come with exacerbations of their CRD.

Each patient had a personal PR exercise record booklet summarizing each exercise, and both during the PR and on completion participants were encouraged to continue with the exercises in their homes.

### Data collection

Qualitative methods were used to collect data before, during, and after PR using individual interviews and ethnographic observations of PR sessions with brief interviews and group discussions.

#### Study population

Participants were selected for interview, using purposive sampling^20^ to ensure inclusion of a range of participants in terms of gender, age, and nature and severity of symptoms.

Ethics and other approvals were obtained from the Mulago Hospital Research and Ethics Committee, number 440 (October 28, 2013) and the Uganda National Council of Science and Technology.

#### Pre-PR interviews

Interviews are commonly used in qualitative research to gain insight into the social world of research participants.^21^,^22^ In this study, it was through talking to patients that it was possible to explore the world of CRD from the perspectives of the sufferers. Semi-structured interviews (10–20 minutes) were conducted using an interview topic guide to gather pre-PR intervention information on patients’ experiences of living with CRD. The interviews also gathered contextual information on general sociodemographic characteristics of the PR participants including age, gender, marital status, level of education, residence, position in the family/community, and employment/occupation status.

#### Ethnographic observation

PR sessions were observed to gain insight into the traditions and behaviors of the PR group.^23^,^24^ These observations facilitated an understanding of the environment and culture of PR; the interactions between group participants (what PR participants said); patterns of behavior (what PR participants did/ought to do); and customs (habits/traditions), and how PR is brought to life as a practice. Data were collected using an observation guide, and field notes were taken. In addition, the opportunity was taken during PR to talk with participants using a topic guide to investigate: the impact of respiratory disease on their lives during PR; identify problems or difficulties caused by attending PR; and gather information on what went well and what did not go well, and any recommendations to improve it.

#### Post-PR follow-up interviews at 6 weeks

Individual interviews were conducted using a topic guide, with participants in their homes 6 weeks following completion of PR. Participants were purposively sampled based on gender, age, severity of condition, responses during pre-PR interview, and ethnographic observations. Participants’ experiences of PR, impact of CRD on their lives following PR, maintenance of PR at home including types of exercises performed, how they were performed, and barriers to maintenance were explored.

#### Focus group discussions

At the end of three PR programs, a group discussion was conducted. Focus groups capitalize on group interaction.^25^ Through talking to one another, patients were able to comment on one another’s experiences of PR. Information was gathered on the extent to which PR exercises met expectations of the participants, problems encountered, perceptions and local understandings of PR, and areas of improvement.

Interviews and group discussions were conducted in the local language (Lugandan) and audio-recorded with the consent of the participants. Where consent to record was not granted, detailed field notes of interviews were taken.

### Data analysis

Analysis focused on the experiences of PR on the lives of patients with CRD in Uganda. The research sought to answer two questions: 1) what are the lived experiences of patients diagnosed with CRD and 2) how does PR alter these experiences? Analysis was performed collaboratively between researchers in Makerere University, Uganda and Plymouth University, UK, to encourage a breadth and depth of analysis from different perspectives and viewpoints.

Data were transcribed in Uganda in the local language, anonymized, and imported into Atlas-ti for analysis. Anonymized data were then translated into English and imported into the QSR NVivo software platform for further analysis.^26^ Data and analyses were shared internationally using a secure file transfer system. Thematic analysis^27^ was conducted across all qualitative data and consisted of familiarization with the data by reading and re-reading transcripts while making memos or reflective notes on the literal content, looking closely at words used by participants, interpreting what the data meant by assigning initial codes/classifications to segments of text, and exploring relationships between these classifications and reducing them to core general themes.

Respondent validation^28^ was adopted whereby feedback of analyses to research participants was provided, and they were invited to judge our interpretations of their experiences, such that they recognized them as their own, or they acted as a correction, or indeed interpretation, of factual errors.

Data sources were triangulated,^29^ which enabled the corroboration of findings with evidence from different sources.

## Results

Forty-two patients with p-TBLD (32 participants) and COPD (10 participants) were recruited to four PR programs. Forty-four semi-structured interviews (10–20 minutes) were conducted with participants: 25 at baseline and 19 6 weeks post-completion. Ethnographic observations and brief interviews were conducted during three PR programs. Three group discussions were conducted on completion of three PR programs.

A number of themes emerged in relation to patients’ lived experiences of CRD prior to PR and how PR altered these experiences.

### What are patients’ lived experiences of CRD prior to PR?

**Figure 1:**
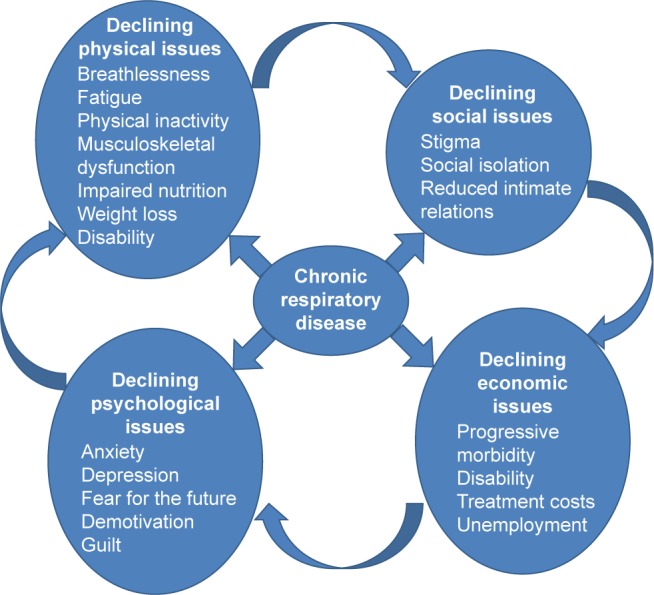
Interactions of impacts of COPD and the vicious circle of CRD progression. **Abbreviation:** CRD, chronic respiratory disease.

Participants reported a range of physical, social, psychological, and economic experiences brought about through living with CRD (Figure 1).

#### Physical symptoms

One example was a 42-year-old male. His breath was constrained and wheezy, and his voice was very low and unclear. He was unable to speak without stopping to cough. When he was asked how he was feeling, he replied:

[…] my body is failing […] my lungs are about to fall out […] I feel smaller, shorter, weak […]. He kept standing throughout the interview and he said he could not sit down because of pain in his chest.

A 17-year-old female had similar challenges:

I faint when in public, so I avoid crowds. I start by feeling dizzy, get out of breath, then I cease to know what follows, then I have to be rushed for an inhaler or an injection. This happens at least twice a week. I also get headaches and chest pain. I have poor appetite and today I have had only half a cup of milk and half a chapatti. When I force myself to eat more, I get nausea.

Even holding a conversation was difficult for this 56-year-old female:

I was treated for TB in 1991 and felt better after completing treatment. But, after some time I got inner chest pain and now I can’t even lift heavy things and can’t sleep on my right side. I feel breathlessness especially when walking uphill. I use a lot of energy to breathe – breathing tires me too, too much. Even speaking – having a conversation with people is a problem.

#### Psychological

The physical impact of CRD also impacted negatively on mood and family relations.

I go out of my moods and I am sometimes unnecessarily harsh to my children. (56-year-old female)

And because of this condition of mine, I get mental lapse and I get irritated so easily. (48-year-old male)

#### Social and intimate relations

Some patients reported that CRD also impacted upon their social and intimate relationships; a 50-year-old male:

When I am in public I cough almost all the time and when people see someone with a prolonged cough most of them start saying that person is sick of TB and TB is contagious, so they discriminate me. I don’t feel free in public at all because of coughing all the time […] even sex, I can’t handle any more. I ask myself is it because of my lung disease? My sex drive is very low, am no longer like I used to be […] sexually I am very weak.

These narratives illuminate how CRD affects the daily lives of sufferers and their families. Participants report that symptoms such as cough, pain, and breathlessness affect mood, the ability to engage in physical activity, appetite, sleep, and social and intimate relationships. In addition to this, what patients know and understand about their lung condition shapes not only their own but also society’s relationship to it.

#### Knowledge and beliefs about CRD

Participants’ experience of CRD was shaped by understanding of the condition and expectations of treatment and prognosis. P-TBLD participants were still perceived to be infectious within Ugandan communities, and therefore, considered to be dangerous and had to be avoided.

The problem is lack of enough information about our conditions. People still think that we can still transmit TB. The fact that we still come to hospital makes it difficult to convince people that we are cured of TB and that we can no longer transmit it. The linkage of TB to HIV/AIDS makes the situation even worse; whoever has TB is always suspected of having HIV/AIDS. (Focus group)

Some participants thought that their condition was due to witchcraft and therefore had to be cured by performing certain rituals and using herbs. Others thought that the solution to their problems was continued medication.

I have swallowed a lot of medicine and am fed up, that’s why I have decided to wait for my day and just burst and die […] we were not being helped by the many medicines that we were taking. (50-year-old male)

However, while medication might resolve an exacerbation of CRD, it does not resolve lung damage, and symptoms of breathlessness persist. A commonly reported expectation was that with treatment, CRD could be “cured,” with complete resolution of the symptoms, disability, and poor prognosis.

I expected my lungs to heal completely and get back like they were before TB. But when I learnt that the status of my lungs will never be reversed, my ambitions in life changed and my focus is now more on the health than anything else. I compare myself to other people and this affects my confidence and decision-making and often I just give up. (47-year-old male)

Such expectations impacted negatively on motivation and support from the community for people with CRD.

The problem is that these (CRD) complications appear in different ways, and therefore attract different interpretations as well as solutions. Some people advise us that we go to pastors for prayers, recommend traditional healers or use of herbs, and others just avoid us all together. (67-year-old male)

Because of a lack of knowledge about their condition, and the often unfulfilled expectation of being “cured,” participants’ conditions were often misunderstood by their family members and the community in general, which negatively impacted on them. Social beliefs, misperceptions, and myths surrounding CRD and related symptoms resulted in lack of confidence and depression, leading to social isolation.

#### Body image

Not only did the knowledge and beliefs of participants and their local community about CRD affect participants’ experience of daily life but also their physical appearance reinforced its impact. Participants reported loss of appetite and weight loss. Ethnographic observations of participants revealed some looking emaciated and miserable, wearing oversized clothes and having reduced mobility.

[…] it is always important for one to think that she or he is normal. But this disease eats your body mass, and everyone notices that there is a problem. I used to be fat and looked smart, but now for example I cannot put on collarless T-shirts because my bones are out. I used to like sportswear and shorts which I can longer wear. My appearance used to maintain some level of confidence in me among my friends, but now I no longer feel as if I am myself. My spouse too thinks that I am handicapped, and she is worried that I may not live as long as I should because my appetite is no longer as good, because my body shrank. (42-year-old male)

The changes reported by this participant affected adversely how he wanted to portray himself to the outside world, altering his sense of identity within the family and society. Physical changes, symptomatology, reduction in physical functionality, emotional well-being, intimate and social relations, all impact on sufferers’ ability to earn a living and provide for themselves and their family.

#### Economic productivity

It is commonly understood that there are added benefits to working besides financial rewards. Symptoms of CRD such as cough, pain, and breathlessness affect patients’ ability to engage in physical activity and manual work, and thus, earn a living.

It has affected me economically because if someone offered me a job I wouldn’t be in position to take it on because of my health condition. I can’t do heavy jobs/work. And because of coughing all the time, I don’t want to socialize so it becomes hard for me to work. (26-year-old female)

I no longer work, when I try to work I feel powerless and my heart beats faster and I get breathlessness. So, I decided to give up work. (44-year-old female)

Furthermore, many patients reported their contribution to their community was reduced, whether paid or not. Such activities included caring for children or grandchildren, caring for animals, and domestic activities.

### Experiences of PR

While PR was a novel treatment in Uganda, recruitment and retention to the program were brisk and patients reported a range of improvements in their lived experience of CRD. PR requires active participation from participants and shifts patients from a passive consumer of medication to an active participant in rehabilitation. It consists of two main elements: education and physical exercises.

#### Education

During the education session, patients learned about CRD: causes, effects, and management. In particular, it encourages the participant to manage their own condition and engage in positive behavior changes, such as increased physical activity, rather than being the passive recipient of treatment. This directly challenged existing and traditional knowledge and beliefs about their condition.

[…] the first thing we learnt was that our condition was not due to witchcraft, that it can never be cured, and that we just have to learn to live with it. We learnt about the effect of gaseous substances – smoke, perfumes, dust, tear gas […] on our health. We now know what to avoid and what to do in case of any problem. Very helpful too was to learn the importance of eating a balanced diet and which foods to avoid. We know how to look after ourselves better. (42-year-old male)

The education program encouraged self-efficacy and created more realistic expectations of the benefits of medication, enabling some patients to realize that, for the most part, they can manage without medication.

It is through the PR exercises that I realized that I can actually live without drugs, herbs, and all sorts of misleading advice by different people. I would rush to a clinic for every small illness, breathlessness, pain … but now I know what to do, and where to go in case the problem persists, and I no longer take medicines anyhow. This helps me to save money and time, which I would spend in clinics and buying medicines. (36-year-old female)

#### Exercises

Patients discovered that, despite scarred and painful lungs, they are able to perform the exercises and improve their health and ability to carry out activities of daily living independently, with a variety of benefits. Within a few weeks, participants reported improvements in breathlessness, strength, and mood. As patients felt improvements in their well-being, they experienced both individual and shared gains with immediate and long-term benefits, including return to work.

Before the (PR) exercises I would spend an hour and a half just washing my face and dressing up, and I would feel so tired just with those two activities and I would rest. Even walking a few meters away, I would come back very breathless as if I have been running. But now I am even thinking of resuming attending to my kiosk since I can walk, stand for long, pain has reduced, and less dependent to others. (56-year-old female)

During the time I was doing the (PR) exercises I did not faint, but when I stopped, I started fainting again, I lost appetite and became weaker, which compelled me to do the exercises again. The positive outcomes gave me hope that my condition would become better. That is why I continued with the exercises. (17-year-old female)

Participants commonly reported that the benefits of exercise on specific symptoms were dependent on continuing the exercises. Participants with p-TBLD often reported chest pain before PR and a major improvement or abolition of the pain after PR. The pain was consistently reported between participants and was described as sharp and severe. The pain tended to be postural, worse on lying down, especially lying on one side and was relieved by turning over. This was a very important problem and severely disrupted sleep.

I used to get too much pain when I would cough or when I got a cough but now, it is no longer that much, okay I get the pain, but it is not as severe as it used to be. (17-year-old female)

When I miss a day without doing the exercises I get general body pains which I actually don’t get when am consistent with my exercise. Otherwise the exercises are going on well and I try to make sure I don’t miss doing them. (64-year-old female)

We saw repeatedly during the study that patients readily engaged with PR. These patients experienced through their own experiments that PR is beneficial to their health and well-being. This served to facilitate the sustainability of PR at home, after completion of the program.

### How does PR alter patients’ experiences of CRD?

#### Physical

Participants reported that PR reduced physical dependency and the burden of care so often placed on family members.

Before PR I would rest several times before completing something, which reduced my role as a husband and father. I could not wash clothes, which forced me into a very embarrassing situation where my daughter had to wash my panties, and I could hardly bath myself, I was dependent on others for so long. But now, I can do all those things and I feel a big difference and most importantly, I can provide for my family. (42-year-old male)

Improved functional capacity brings with it added benefits of increasing independence, regaining the ability to undertake most activities of daily life on their own.

#### Psychological

Where previously patients felt self-pity, following PR they regularly reported improvement in their sense of value.

I no longer have self-pity on myself, this they taught us during the education sessions in the PR program. (53-year old female)

Improved self-worth following PR brought about positive social and intimate relationships for many patients.

#### Social and intimate relationships

The benefits of PR were also reflected in intimate relationships.

What gave me hope was the fact that I regained my manhood. Before I took part in the PR program, I was not able to perform my conjugal duties and even my wife was complaining about it. Regaining my sexual potency was the first vivid indicator to me that there would be significant changes in my health and life in general. (46-year-old male)

This patient is not only able to take his place as the man in the home but also feels he has almost returned to his pre-disease state, rising early and swiftly doing his work.

I feel I am now a man in the home. I wake up at 6.00 am and do my work throughout the day. I am almost the way I was before getting the disease [TB]. Sometimes my colleagues tell me to slow down on my activities, otherwise I feel no problem at all. (42-year-old male)

#### Economic

Improved physical capacity and social relations brings about the possibility of returning to work or education, enabling patients to earn a living and re-engage with the local community.

I have a stall in the market where I sell Irish potatoes, vegetables, onions, tomatoes etc. I go very early in the morning to the bigger markets to buy stuff which I then sell at my stalls. I also make paper bags which I sell and earn money, I make candles, mats and bags. The bags I make them from beads. Before the rehab, I used to feel a lot of pain to do the above activities especially making mats. I even teach other people the above skills and they pay for my services. I am also a volunteer at TASO, I help those who are just starting ART. I encourage them and tell them about adherence and how to take their drugs … I no longer suffer with pill count. I used to take too many drugs … recently I did not feel well one night but because of the PR program I know exactly what to do. I remembered that they taught me to always breathe in and out twice if I get such a thing and when I did it twice, I felt better. So, I learnt what to do for my health condition. (60-year-old female)

Not only is this patient earning money but she is also teaching and volunteering, both of which build interpersonal skills, benefits the community, reduces social isolation, increases social connectedness, builds friendships, and improves self-esteem and mental health. She has developed the knowledge and confidence to manage her symptoms using the tools of PR, without automatic recourse to medication, which is costly.

#### Group support

The benefits of group PR are abundant and include a supportive network of like-minded people who have shared goals in an environment for sharing common experiences, providing help with commitment and motivation. PR participants valued each other as co-supporters and educators. When this participant was struggling with her appetite, the group encouraged her to regain her appetite:

We are now like brothers and sisters – the exercises have brought us together and made us feel for one another. When I missed a meal for almost a week due to lack of appetite, everybody [in the PR group] – young, old, male, female was concerned. Each of them was advising me on what to do, especially encouraging me to eat something. We now have each other’s telephone numbers and can call to support each other. (22-year-old female)

PR participants shared information about limitations of medications in relation to their health conditions, types of exercises and weights that were effective, advised each other on how to improve their appetite, improve their appearance, have hope, and management of their life in general. This social network was an important contributor to the continued attendance and improvement in moods and social isolation.

#### Maintenance

Not only did patients ensure they did not miss a PR session, through a process of improvisation, they maintained the exercises at home.

[…] what I do at my home is to use paving bricks. I broke them into pieces which I combine to get the appropriate weights I need. I also use 5–10 L jerrycans, and water bottles as weights for my exercises. (67-year-old male)

The equipment used in the program – weights, static bicycle, and steps could not be obtained in local shops. Patients commonly used water bottles or carriers as weights.

#### Community benefits

PR participants also became a resource for their communities, giving advice particularly on seeking appropriate care for CRD and related complications. They also shared information on symptoms and how to prevent CRD and became points of reference regarding TB, cough, and other chest-related illnesses in their communities. One of them described their identity and new roles in society:

Improvement in our health conditions has in some ways changed the perception of TB and related conditions and has generated confidence in the community. People come to inquire about CRD, and we are now a point of reference and source of information on TB. We advise communities about sources of care, and we share information on how to prevent CRDs. We are now “experts” in our own way. (Focus group PR participant)

This transformation from being a powerless victim of an illness to be an expert helping others was a major boost to self-confidence and a significant benefit to other sufferers and their community.

### Summary of the themes

Patients reported the profound impact of being breathless on behavior (avoiding exercise) leading to deconditioning, weakness, and fatigue, which in turn led to impairment of activities of daily living (Figure 1). Stigma associated with persistent cough and breathlessness and perceived risk of contamination caused isolation within the family and society. Consequent depression and anxiety further reduced activity, contribution, and economic activity. Reduced income reduced self-esteem, affected the family, and often led to food shortage. The resulting poor nutrition aggravated weakness and weight loss related to the lung disease.

**Figure 2:**
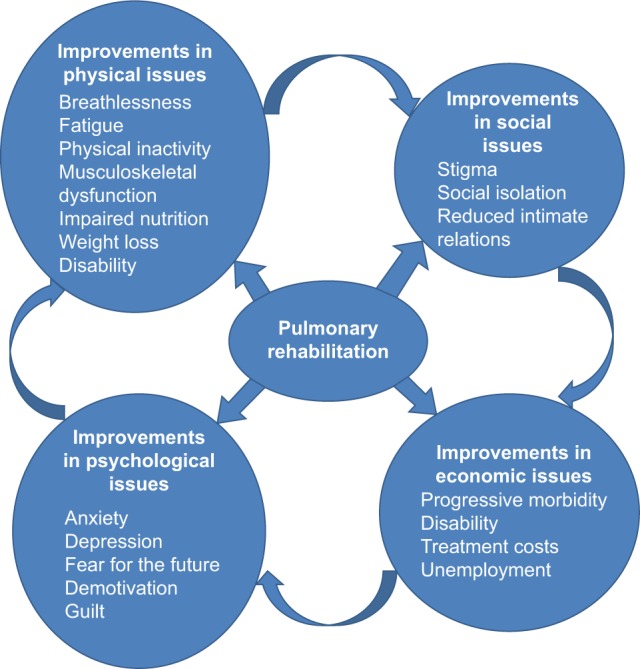
The improvements brought about by pulmonary rehab on the impacts of COPD and the vicious circle of CRD progression. **Abbreviation:** CRD, chronic respiratory disease.

Happily, PR reversed these trends by improving fitness, breathlessness, and dependency, so social and economic activity increased, and moods and self-esteem improved (Figure 2).

## Discussion

This qualitative study sought to explore the lived experiences of patients diagnosed with COPD and how PR alters these experiences. In an extensive series of qualitative interviews, ethnographic observations with brief interviews, and focus groups, we found that PR improved a wide range of physical, social, psychological, and economic outcomes in participants.

The rising morbidity and mortality of CRDs in Africa, and their attendant physical, social, psychological, and economic consequences places considerable strain on patients, healthcare, and society.^6^,^30^,^31^ The cycle of decline in people with COPD is well established in high income countries (HICs); breathlessness causes reduced activity and consequent physical deconditioning, demotivation, and leads to major psychosocial impacts.^15^,^32^ In this study, we included patients with both COPD and p-TBLD. In Uganda, TB is prevalent;^33^ we found that patients with persistent respiratory symptoms were stigmatized as previously described in India, impairing social participation.^12^ Family and community members were fearful that the condition could be contagious; this occurred in COPD patients, but was most prominent in the experiences of those with previous TB. The fear of infection causes social isolation. Local health beliefs about the cause, such as witchcraft, made matters worse for the patients and those close to them. Thus, in Africa, we found that the negative psychosocial impacts of CRD were greatly magnified compared to the experiences of patients with CRD in HICs.

Poverty also added to the problems faced by people with CRD. In 2013, approximately 35% of households lived below the international extreme poverty line of just $1.90 per day.^34^ Uganda has a very high level of unemployment, estimated to be around 90% in the over 25 years old and 66% of the employed population works in agriculture, producing food.^35^ These jobs tend to be manual in nature, requiring a degree of physical fitness beyond the ability of most people with CRD. Inability to perform manual labor in an economy dominated by such work, impacts on poverty. At the same time, increased costs for medicines and consultations (many of which are ineffective) increase financial strains when earnings are reduced. Lack of food and medicines accelerate the physical decline. CRDs affect the poorest people in poorest societies and conspire to worsen their poverty.

PR allowed participants disabled by CRD to get back into work with all the attendant benefits of employment. We found out that many patients were wasting scarce money on ineffective treatments and that PR provided education on the true causes of CRD and useful relief of symptoms.

To address the rising tide of long-term conditions requires long-term solutions, such as the chronic disease model.^36^ Central to these solutions are improved training, especially in primary care, early diagnosis, patient education to adopt healthy lifestyles, self-management of acute exacerbations, and affordable accessible medication and rehabilitation services.^37^ While the treatment costs of COPD in HICs are very high and rising (for example, the USA spends $50 billion annually),^38^ by contrast, PR is a highly effective but relatively cheap intervention using only local resources, and it is scalable and sustainable.

In this study, we show that PR has the potential to restore the physical, social, psychological, and economic functioning of patients with CRD, and this is in-line with other qualitative studies from Western countries.^39^,^40^ PR addresses all the elements in the vicious cycle described in Figure 1. PR was acceptable, feasible, and produced strong evidence that incapacitation can be reduced and functioning improved. The impact of PR was at times life-transforming. No serious disadvantages were reported by patients. The challenge with PR is to implement it when the focus of health systems is to treat the acute illness, not the underlying cause.

Patients moved from being inactive patients in the PR program to become self-motivated and supportive participants, and also co-educators. At community level, PR participants were instrumental in increasing levels of awareness about CRD and PR in the general population, promoting prevention, and changing treatment-seeking behavior. Participants became symbols of not only a successful medical intervention but also points of reference regarding TB, cough, and other respiratory and chest-related illnesses in their communities. This increased knowledge worked to integrate them back into a society from which they had previously felt ostracized, a feature of PR in other settings.^41^

Limitations include that this was conducted in a single center with a relatively small sample of patients and findings may not be generalizable to the wider population. In addition, this was a purposive sample and may have been biased to people responding positively. In a culture where deference and respect for the medical establishment is high, participants may have felt pressured to respond positively about PR. However, quantitative outcome measures show clinically important improvements in quality of life, exercise capacity, and respiratory outcomes as a result of PR, and a high retention rate of participants; 85% completed the programme.^19^ The data collection in this paper were collected pre-PR and around 6 weeks after completion of the program; further longitudinal study is required to evaluate the long-term impacts of PR in this setting. We are currently conducting research on implementing PR in Crete, Vietnam, and Kyrgyzstan.

## Conclusion

Our study suggests that people with CRDs have a complex array of interacting problems including symptoms, disabilities, and psychosocial impacts with major negative effects on them, their families, their community, and health services. Unlike any other intervention, PR addresses all of the major problems faced by people with CRDs and is acceptable to patients. For some patients, the program was life-transforming. PR is a low-cost intervention but needs to be adapted to the patients, culture, and systems of Uganda to work well.
